# Determinants of maxillary canine impaction: Retrospective clinical and radiographic study

**DOI:** 10.4317/jced.54095

**Published:** 2017-11-01

**Authors:** Michele Laurenziello, Graziano Montaruli, Crescenzio Gallo, Michele Tepedino, Laura Guida, Letizia Perillo, Giuseppe Troiano, Lorenzo Lo Muzio, Domenico Ciavarella

**Affiliations:** 1 University of Foggia, Department of Clinical and Experimental Medicine; Foggia,Italy; 2 University of Aquila, Applied Clinical Sciences and Biotecnology, Aquila, Italy; 3 Second University of Naples, Department of Dentistry, Orthodontic and Surgical; Naples, Italy

## Abstract

**Background:**

The aim of this study is to evaluate determinants of maxillary canine impaction taking into account both canine position related variables and the pattern of facial growth.

**Material and Methods:**

A retrospective clinical and radiographic analysis was carried out on 109 patients aged between 9 and 10 years at the time of first evaluation. At baseline, SN-GoMe angle, the interincisal angle, the canine angle α and the canine distance d were used to characterize canine location and vertical facial growth. At the end of a two years follow up period the eruption state of each canine of each patient was recorded and accordingly classified as erupted or impacted on a clinical and radiographic basis. Univariate and multivariate statistical analyses were performed, including correlation among the studied variables and principal components analysis; several machine learning methods were also used in order to built a predictive model.

**Results:**

At the end of the two years follow up period after the first examination, 54 (24.77%) canines were classified as impacted. Except for Angle α values, there were no statistically significant differences between impacted and erupted canines. The studied variables were not significantly correlated, except for the SN-GoMe Angle and the distance d in the impacted canine group and the angle α and the distance d in erupted canines group. All variables, except for SN-GoMe Angle in erupted canines, have a partial communality with the first two principal components greater than 50%. Among the learning machine methods tested to classify data, the best performance was obtained by the random forest method, with an overall accuracy in predicting canine eruption of 88.3%.

**Conclusions:**

The studied determinants are easy to perform measurements on 2D routinely executed radiographic images; they seems independently related to canine impaction and have reliable accuracy in predicting maxillary canine eruption.

** Key words:**Canine impaction, Determinants, Facial growth.

## Introduction

The pathogenesis of maxillary canine impaction, a common (1,2) and clinical challenging dental condition, can be related to genetic and anatomical factors. In fact, two theories have been proposed: the genetic theory (3) and the guide theory (4-6). The former seems to be sustained by the observation that maxillary canine impaction is often associated with other genetic conditions such as: facial cleft, skull and facial syndromes, other congenital alterations of shape and number of teeth (2,7,8). The latter attributes canine impaction to the lack of an eruptive guide supported by the lateral incisor (7), because of agenesis of the lateral incisor (9), and/or lateral incisor malformation (i.e. conoid or microdontic lateral incisor) (10). In addition, it has been reported that a number of other conditions may favor canine impaction: e.g. the presence of odontoma (11,12), the permanence of the ankylosed deciduous canine (13), the premature appearance in the dental arch of second molars or the reduced bone development in the canine area, retention of other dental elements (14). Recently, it has been suggested that the risk of canine impaction could be also associated to the pattern of facial development (10,15); it has also been reported an increased risk of canine impaction in patients with deep bite (14,16).

Traditionally, a series of geometric measurements made on radiographs have been indicated as predictors or maxillary canine impaction (17).

The aim of this study is to evaluate determinants of canine impaction, by measuring a number of variables on both panoramic and lateral head films, thus taking into account both canine position and the pattern of facial growth, and elaborate a model capable to predict the risk of impaction on a case by case basis.

## Material and Methods

A retrospective clinical and radiographic analysis was carried out on 109 patients (44 males and 67 females: mean age 9.34 years) followed-up at the School of Dentistry of Foggia University (Italy) and at the Second University of Naples (Italy). Written informed consent was provided by patients’ parents for the enrolment in the study.

Inclusion in the study was performed according to the following criteria: i) age between 9 and 10 years at the time of first evaluation; ii) complete orthodontic evaluation, including casts of maxillary and mandibular dental arches, photographs, panoramic and lateral-head radiographs at the first evaluation; iii) physiologic upper canines retention at the first evaluation; iv) no orthodontic treatment in the two years period following the first examination; v) clinical and radiographic re-evaluation after this period. On the other hand, exclusion criteria were: i) presence of destructive dental caries; ii) unilateral crossbite; iii) central incisors trauma; iv) conoid lateral incisors; v) congenital abnormalities and alterations in the development, shape and number of teeth.

For all patients and for each maxillary canine, data described in  Table 1 were registered at the time of the first examination in order to identify potential parameters associated with the lack of upper canines eruption. In particular, SN-GoMe angle was used to assess the pattern of vertical facial growth; the interincisal angle was used as a measure of the crowding of the maxillary anterior region; the angle α and the distance d were used to characterize canine location within the jawbone.

**Table 1:**
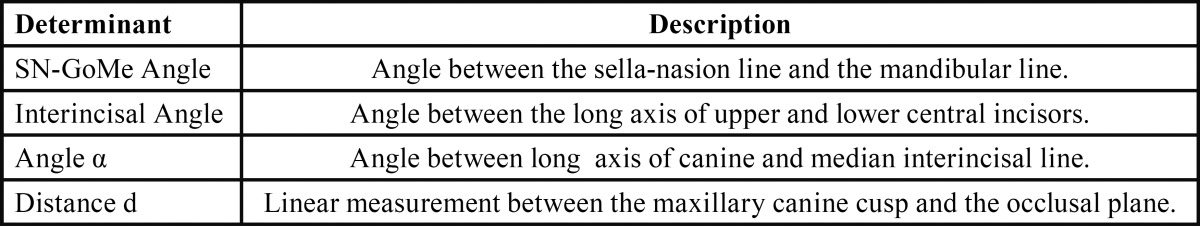
Dental and cephalometric measurements performed on radiographs (panoramic and lateral-head films, respectively) taken at the first examination.

At the end of the two years follow up period after the first examination, the eruption state of each canine of each patient was recorded and accordingly classified as erupted or impacted on a clinical and radiographic basis. In particular, were considered as impacted canines whenever the following conditions occurred: corresponding deciduous canine was still in place, no space for the permanent canine, complete formation of permanent canine root. The above mentioned data of 218 maxillary canines were collected and organized in a digital sheet.

Univariate and multivariate statistical analyses were performed by Wolfram Mathematica ® v. 10 software. The single maxillary canine was used as statistic unit. Every canine was annotated with information regarding the above specified data; thus, a matrix including values of all  Table 1 variables was constructed. Normality test was performed by means of X2 test. Unpaired t- or u-test (according to normality distribution and with a significance level of 5 %) were used to analyse statistically significant differences of median values of investigated variables between erupted and impacted canines.

Correlation among the studied variables was investigated in erupted and impacted canines by calculating the Spearman rank correlation coefficient (r); Student’s t test with n-2 degrees of freedom was used to test whether the calculated values of r were significantly different from 0 (*p*<0.05). The r2 coefficient was also calculated in order to provide a measure of the common variance between two variables, that is to say the proportion of variance accounted for in one of the variables or “explained” by the other.

Principal component analysis (PCA) was performed in order to identify, for each of the studied group (i.e. erupted and impacted), the principal and most variable components and their correlation with all the studied variables. Variables exhibiting at least 0.5 communality values were taken into account.

In addition, several machine learning methods were used to classify samples according to the following conditions: erupted vs impacted. In particular, the following methods were used: random forest, support vector machine, neural network, nearest neighbors, naive bayes, logistic regression; results from the classifier showing the best performance were taken into account. Classification between erupted and impacted canines was performed by randomly creating one training set (174 records: 131 erupted and 43 impacted) and one validation set (43 records: 32 erupted and 11 impacted). A bootstrap resampling procedure was used to verify whether the variables were truly independent predictors or, rather, were noise variables.

## Results

At the end of the two years follow up period after the first examination, 164 (75.23%) canines erupted normally, whereas 54 (24.77%) canines were classified as impacted.

Data of the studied variable are detailed in  Table 2. It is evident that, except for Angle α values, there were no statistically significant differences between impacted and erupted canines.

**Table 2:**
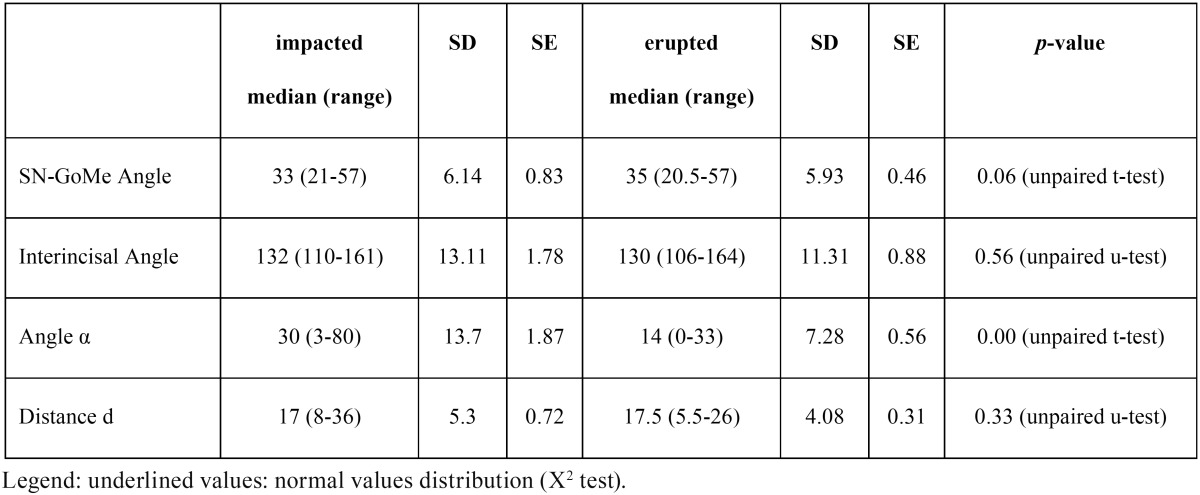
Descriptive statistics of studied variables in impacted and erupted canine groups.

Correlation coefficients of the studied variables for impacted and erupted canines are shown in  Table 3 and  Table 4, respectively.

**Table 3:**
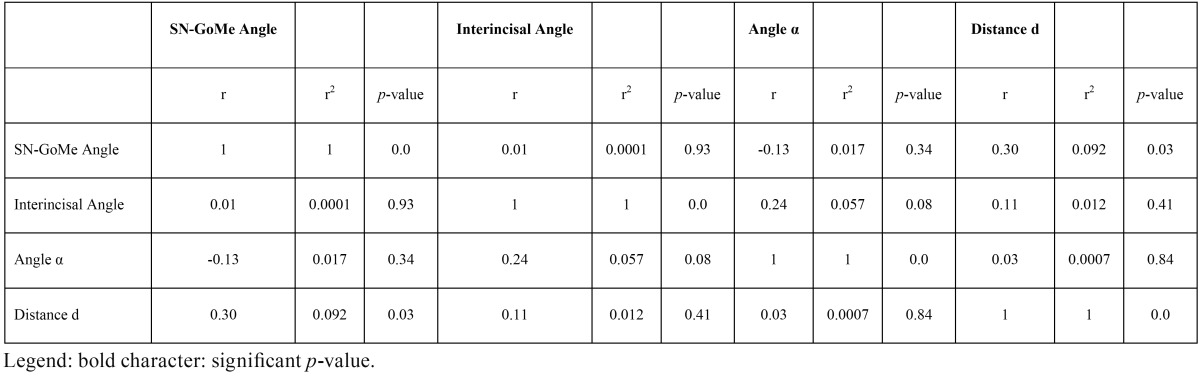
Correlation of studied variables in impacted canines.

**Table 4:**
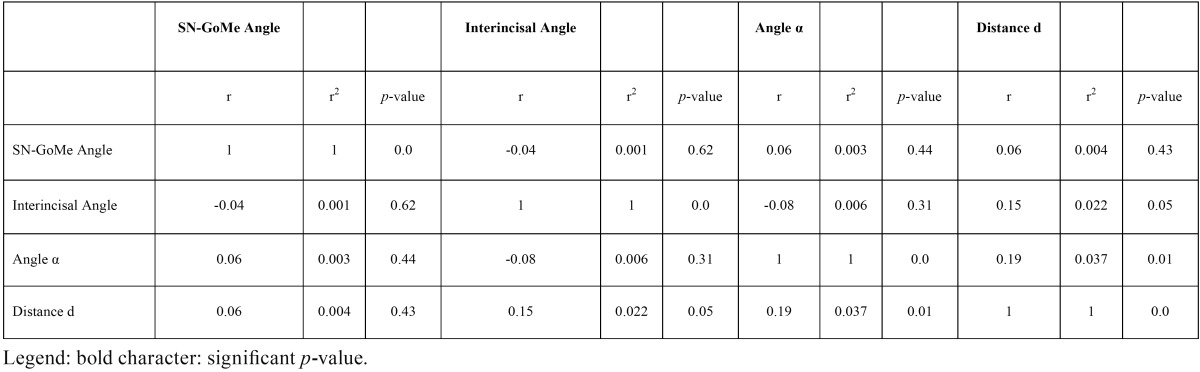
Correlation of studied variables in erupted canines.

The studied variables are not significantly correlated, except for the SN-GoMe Angle and the distance d in the impacted canine group and the angle α and the distance d in erupted canines group. Nonetheless, in both instances the positive correlation is weak (0.30 and 0.19, respectively).

PCA analysis showed that in both impacted and erupted canines groups there were two principal significant components explaining 63.03% and 58.28%, respectively, of the total variance. Component matrix with details of partial communality values and correlation coefficients between the investigated variables and the first (PC1) and the second (PC2) principal component are shown in  Table 5.

**Table 5:**
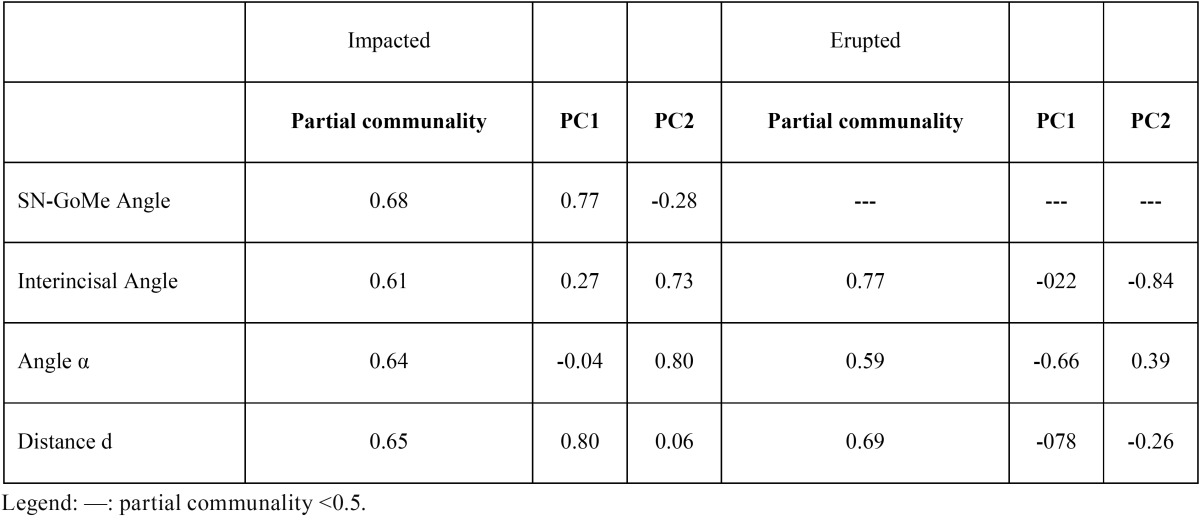
Component matrix of the first two principal components and the investigated variables.

All variables, except for SN-GoMe Angle in erupted canines, have a partial communality with the first two principal components greater than 50%, thus, confirming their potential impact in determining upper canine impaction and their independence from each other.

**Figure 1:**
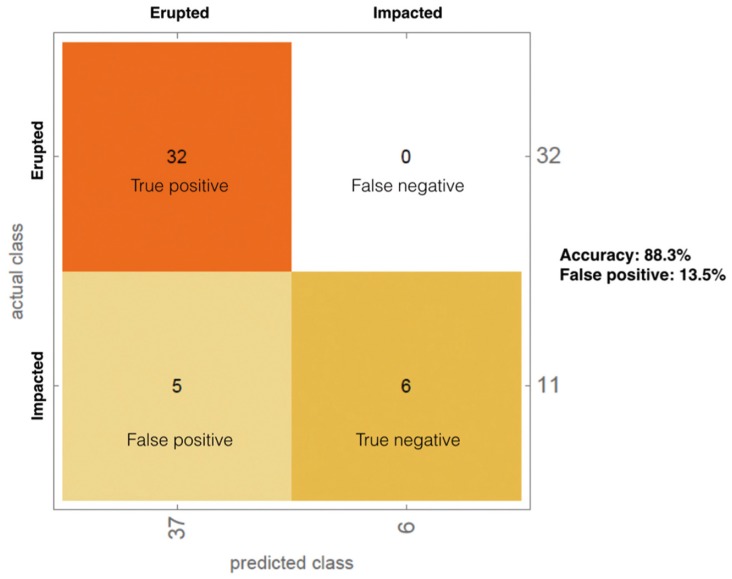
Classification of erupted vs impacted by random forest methods.

Among the learning machine methods tested to classify data, the best performance was obtained by the random forest method, with an overall accuracy in predicting canine eruption of 88.3% (Fig. 1).

## Discussion

Maxillary canine impaction may cause detrimental effects on jawbones development, occlusion stability and adjacent teeth (e.g. root resorption). In addition, its treatment is multidisciplinary (i.e. combination of surgical and orthodontic intervention), often associated with prolonged treatment time (18) and a reduction of treatment success according to increasing patient’s age (19). Thus, it is clinically sensitive the possibility to predict impaction and establish proper interceptive treatment. Several studies have investigated the possible predictors of canine impaction and orthodontic treatment choices with 2D radiographs (20,21). Recently, the use of 3D images obtained by means of cone-beam computed tomography (CBCT) has been also reported (17). In the present study we have used a number of potential determinants of maxillary canine impaction measured on 2D images, in particular panoramic and lateral head radiographs, because these are simple and easy to perform x-ray investigations routinely used in screening and treatment planning of malocclusions, especially in children. As regards the selection of potential determinants, basing on the etiopathogenetic theories summarized in the introduction section, we have chosen variables accounting for both canine position and facial growth pattern. The only determinant (22,23) which was significantly different in erupted and impacted canines was the Angle α; in fact, impacted canines were in a significantly more “horizontal” position. Nonetheless, the correlation analysis among the studied determinants, as well as PCA confirms that the four selected determinants are:

i) almost independent; in fact, very weak correlation were found only between SN-GoMe Angle and the distance d in the impacted canine group and the Angle α and the Distance d in erupted canines group;

ii) capable to explain, to a high extent, the canine impaction, according to the communality values with the principal components responsible of such condition. In other words, the principal two and statistically significant components explaining 63% of impaction are explained from 61% to 68% by every single investigated determinant (see  Table 5 for details). It is worth noting that, under this point of view, the SN-GoMe Angle is the most relevant determinant in explaining canine impaction, but it is not significatively associated with canine eruption. Thus, it seems that the pattern of facial vertical growth is an meaningful variable to be considered in assessing the risk for maxillary canine impaction.

Clinical significance of investigated determinants is further underlined by their efficacy in predicting the possibility of canine eruption or, conversely, the risk of impaction, as confirmed by our predictive model obtained by instructing a learning method machine (i.e. random forest) on our data series, which is characterized by a valuable 88.3% of overall accuracy.

## Conclusions

The studied determinants (i.e. SN-GoMe Angle, Distance d, Angle α and Interincisal angle) are easy to perform measurements on 2D routinely executed radiographic images; they seems independently related to canine impaction and have reliable accuracy in predicting maxillary canine eruption eruption/impaction.
